# Factors affecting the growth of children till the age of three years with overweight whose mothers have diabetes mellitus: A population-based cohort study

**DOI:** 10.1186/s12887-021-02768-z

**Published:** 2021-07-02

**Authors:** Yuan-Der Huang, Yun-Ru Luo, Meng-Chih Lee, Chih-Jung Yeh

**Affiliations:** 1 Department of Public Health, Chung-Shan Medical University, Taichung, Taiwan; 2 Department of Obstetrics and Gynecology, Chia-Yi Hospital, Ministry of Health and Welfare, Chia-Yi, Taiwan; 3 Department of Family Medicine, Taichung Hospital, Ministry of Health and Welfare, Taichung, Taiwan; 4 Institute of Population Health Sciences, National Health Research Institutes, Miaoli, Taiwan; 5 College of Management, Chaoyang University of Technology, Taichung, Taiwan

**Keywords:** Birth cohort, Maternal diabetes, Childhood overweight/obesity, Growth

## Abstract

**Background:**

The prevalence of diabetes mellitus (DM) during pregnancy and childhood obesity is increasing worldwide. Factors affecting the growth of children with overweight whose mothers had DM are complicated and inconclusive. Few longitudinal studies have focused on the growth of infants with macrosomia born to mothers with DM and the factors influencing their overweight. This study explored risk factors for childhood overweight/obesity (OWOB) among children of mothers with DM. Perinatal, maternal socio-demographic, infant care, and maternal body weight characteristics as well as child growth until age 3 years were analyzed using a longitudinal design.

**Methods:**

In total, 24,200 pairs of mothers and their children from the Taiwan Birth Cohort Study were included. Combined Taiwan Children Growth Curve report classifications were analyzed for infant growth at birth and at 6, 12, 18, 24, and 36 months old (m/o). A multiple logistic regression analysis with different model settings was used to assess factors affecting the growth of high birth weight children of mothers with diabetic mellitus (HODM).

**Results:**

Children in the HODM group had a higher average body weight than did those in the non-DM group at different age stages. Relative to the non-DM group, weight gain in the HODM group was slower before 18 m/o but faster from 18 to 36 m/o, particularly after 24 m/o. Maternal DM was a major risk factor for childhood OWOB (odds ratio [OR]: 3.25–3.95). After adjustment for related confounders, the OR was 2.19–3.17. Maternal overweight or obesity and higher gestational weight gain were greater risk factors for childhood OWOB at 3 years old after adjusted maternal DM and other selected confounders (OR: 1.45 and 1.23, respectively). Breastfeeding until 6 m/o was a protective factor against childhood OWOB (OR: 0.95). The HODM and non-DM groups did not differ significantly in perinatal, maternal socio-demographic, or infant care characteristics.

**Conclusions:**

Maternal DM is a major factor of childhood OWOB. Maternal body weight before and after pregnancy affects childhood OWOB, and this effect increases with the child’s age.

## Background

The prevalence of diabetes mellitus (DM) is increasing rapidly worldwide and is the most common complication of pregnancy, affecting up to 10% of expectant mothers [1]. In Taiwan, the prevalence of diagnosed DM in pregnancy increased from 2.0% in 1996–1998 [2] to 3.5% in 2001–2008 [3]. A Taiwanese study demonstrated that morbidity is higher among children whose mothers had DM than among children of mothers without DM, and overweight newborns had an increased risk of hospitalization and repeat admission [2].

Infants exposed to high glucose levels prenatally have an increased risk of long-term adverse outcomes, including childhood overweight and obesity (OWOB). Many risk factors contribute to childhood overweight. Maternal obesity is a known major risk factor for childhood obesity. Many studies have demonstrated that mothers with higher body mass index (BMI) are more likely to have overweight infants [4]. Although high maternal BMI is associated with a substantially increased risk of gestational DM (GDM) [5], the causal associations of childhood overweight with maternal DM and obesity remain unclear. In addition to genetic and DM factors of child obesity, the effects of family lifestyle [6], breastfeeding, parents’ education level, and even whether mothers who are able to perceive their children’s body weight status accurately have been discussed [7, 8].

To our knowledge, no studies examining the association between maternal DM and childhood OWOB have considered all potential key confounders, including maternal obesity, gestational weight gain, maternal and infant lifestyle, and care factors. Women with preexisting DM have higher risks of preterm labor, congenital anomalies in their children, adverse perinatal complications, and slowed infant growth in the future. Studies that have not excluded infants with low birth weight born to mothers with DM, especially infants born to women with GDM and type 2 DM, have methodological limitations in their analyses of childhood obesity [9]. A study recommended that infants of women with DM be targeted specifically for obesity prevention [10]. By controlling for maternal BMI, our study compared high birth weight of diabetic mothers (HODM) with children whose mothers did not have DM to explore risk factors for subsequent childhood OWOB and growth. The results may provide information for mothers with the most severe DM to prevent childhood obesity in their children.

## Methods

### Data source and study population

Data used in this study were collected from the Taiwan Birth Cohort Study (TBCS). TBCS is a national population-based cohort study in Taiwan to investigate the health determinants of this 2005–2006 birth cohort at their newborn, infant, child, adolescent, and adult stages, from the life-course perspective. Formal first wave (6 m/o) survey was completed in 2005–2006, followed up in 2006–2007 (18 m/o), 2008 (3 y/o), 2010–2011 (5 y/o), 2013–2014 (8 y/o), and 2017–2018 (12 y/o). The TBCS cohort was expected to follow up till 21 y/o (year 2026–2027). Of the 206,932 live births in the Taiwan birth registration database for 2005, 24,200 pairs of mothers and their children were sampled in the study from the TBCS through a multistage stratified systematic sampling method [11]. This dataset was compiled from the Millennium Cohort Study (MCS) [12] and the National Children’s Study (NCS) [13]. The mothers provided written consent to participate, and this study was approved by the Medical Ethics Committee and Data Protection Board in Taiwan. A total of 21,248 mother–child pairs (87.8%) completed the first wave survey when child’s age is 6 months old. A total of 20,645 mother–child pairs were enrolled into our study. Of the 20,645 pairs who complete the first wave of study, 19,597 (92.22%, 18 months) and 19,344 (91.03%, 36 months) completed the second wave and the third wave, respectively. Our study excluded multiple pregnancies (561 cases) and mothers who did not know their diagnosed DM during pregnancy at the time of interview (43 cases). Children with major or minor congenital fetal anomalies (998 cases) were not calculated into multiple logistic regression analysis models.

### Variable definitions

Children were classified into four groups: children born to mothers without DM (non-DM), Offspring of mother with Diabetic Mellitus (ODM) with high birth weight (>4000 g, HODM), ODM with appropriate birth weight (2500–3999 g, AODM), and ODM with low birth weight (<2500 g, LODM). OWOB was defined as body weight above the 85th percentile at 6, 18, 24, or 36 m/o (separated by sex) according to Taiwan Children Growth Curve report classifications [14].

Mothers were asked questions regarding awareness of diagnosed DM by doctors during pregnancy by trained interviewers (yes or no), maternal BMIs before pregnancy and 6 months after delivery (calculated based on the data reported from the first wave survey), nationality, educational level, area of residence (urban or rural), family income, whether they breastfed or staple fed their children at 6 m/o, and main daytime caregiver (mother or others). Maternal age was defined as mother’s age at delivery (older or younger than 35 years). APGAR scores were classified as more or less than 7. Maternal body weight gain during pregnancy was divided into three categories: <10, 10–14, and >14 kg. Prepregnancy and postpartum BMI were divided into three categories: <18.5, 18.5–25, and >25 kg/m^2^.

### Statistical analyses

We classified the variables of influential factors into four major categories, namely perinatal condition, maternal socio-demographic characteristics, infant care factors, and maternal body weight characteristics. Categorical variables were analyzed using the chi-square test among the non-DM, HODM, AODM, and LODM groups. Fisher’s exact test was conducted if necessary (Table 1).

**Table 1:** Maternal socio-demographic, perinatal care and children’s characteristics in Taiwan

	Non-DM^1^	HODM^2^	AODM^3^	LODM^4^	*p*-value^a^	*p*-value^b^
	(*n* = 20,199)	(*n* = 39)	(*n* = 382)	(*n* = 25)		
**Perinatal conditions**						
Sex					NS	NS
Male	10,566 (52.3%)	19 (48.7%)	223 (58.4%)	11 (44.0%)		
Female	9633 (47.7%)	20 (51.3%)	159 (41.6%)	14 (56.0%)		
Prematurity (<37 weeks)					<0.001^*^	NS
Yes	1387 (6.9%)	5 (12.8%)	39 (10.2%)	19 (76.0%)		
No	18,812 (93.1%)	34 (87.2%)	343 (89.8%)	6 (24.0%)		
Apgar score(1 min)					<0.001^*^	NS
≥7	19,818 (98.1%)	37 (94.9%)	375 (98.2%)	16 (64.0%)		
<7	381 (1.9%)	2 (5.1%)	7 (1.8%)	9 (36.0%)		
Apgar score(5 min)					0.0032	NS
≥7	20,148 (99.7%)	39 (100.0%)	381 (99.7%)	24 (96.0%)		
<7	51 (0.3%)	0 (0.0%)	10 (0.3%)	1 (4.0%)		
Birth defect					<0.001^*^	NS
Yes	960 (4.8%)	2 (5.1%)	32 (8.4%)	4 (16.0%)		
No	19,239 (95.2%)	37 (94.9%)	350 (91.6%)	21 (84.0%)		
**Maternal socio-demographic characteristics**						
Maternal age					<0.001^*^	<0.001^*^
≤35 y/o	18,448 (91.3%)	28 (71.8%)	304 (79.6%)	22 (88.0%)		
>35 y/o	1751 (8.7%)	11 (28.2%)	78 (20.4%)	3 (12.0%)		
Maternal nationality					<0.001^*^	NS
Taiwanese	17,446 (86.4%)	35 (89.7%)	365 (95.5%)	24 (96.0%)		
Non-Taiwanese	2753 (13.6%)	4 (10.3%)	17 (4.5%)	1 (4.0%)		
Maternal education					0.0049	NS
Less than high school	3021 (15.0%)	3 (7.7%)	36 (9.4%)	2 (8.0%)		
High school	8085 (40.1%)	16 (41.0%)	139 (36.5%)	10 (40.0%)		
College and above	9061 (44.9%)	20 (51.3%)	206 (54.1%)	13 (52.0%)		
Family income (NTD)					0.0065	NS
<50,000	8499 (42.2%)	14 (37.8%)	121 (31.8%)	11 (44.0%)		
50,000–150,000	11,020 (54.7%)	22 (59.5%)	246 (64.6%)	14 (56.0%)		
≥150,000	614 (3.0%)	1 (2.7%)	14 (3.7%)	0 (0.0%)		
Living area					<0.001^*^	NS
Urban	9529 (47.2%)	23 (59.0%)	222 (58.1%)	14 (56.0%)		
Rural	10,670 (52.8%)	16 (41.0%)	160 (41.9%)	11 (44.0%)		
**Perinatal care**						
Breastfeeding at 6 m/o					0.0912	NS
Yes	16,611 (82.2%)	33 (84.6%)	307 (80.4%)	25 (100.0%)		
No	3588 (17.8%)	6 (15.4%)	75 (19.6%)	(0.0%)		
Non-staple foods feeding before 6 m/o					NS	NS
Yes	18,148 (89.9%)	37 (94.9%)	347 (90.8%)	22 (88.0%)		
No	2043 (10.1%)	2 (5.1%)	35 (9.2%)	3 (12.0%)		
Main caregiver at daytime (at 1–6 m/o)					NS	NS
Mother	9961 (49.3%)	20 (51.3%)	193 (50.5%)	11 (44.0%)		
Others	10,238 (50.7%)	19 (48.7%)	189 (49.5%)	14 (56.0%)		
**Maternal body weight**						
Pre-pregnancy BMI (kg/m^2^)					<0.001^*^	<0.001^*^
<18.5	4236 (21.0%)	1 (2.6%)	41 (10.7%)	3 (12.0%)		
18.5–25	14,040 (69.5%)	20 (51.3%)	249 (65.2%)	15 (60.0%)		
>25	1923 (9.5%)	18 (46.2%)	92 (24.1%)	7 (28.0%)		
Postpartum BMI (at 6 m/o)					<0.001^*^	<0.001^*^
<18.5	158 (0.8%)	0 (0.0%)	5 (1.3%)	0 (0.0%)		
18.5–25	7330 (36.3%)	0 (0.0%)	83 (21.7%)	7 (28.0%)		
>25	12,711 (62.9%)	39 (100.0%)	294 (77.0%)	18 (72.0%)		
Gestational weight gain (kg)					NS	NS
<10 kg	3401 (16.8%)	6 (15.4%)	85 (22.3%)	6 (4.0%)		
10–14 kg	7919 (39.2%)	13 (33.3%)	146 (38.2%)	8 (32.0%)		
>14 kg	8879 (44.0%)	20 (51.3%)	151 (39.5%)	11 (44.0%)		

^1^Non-DM: newborn born from non-DM mothers

^2^HODM: Newborn born from DM mothers and birth weight more 4000 g

^3^AODM: Newborn born from DM mothers and birth weight within 2500–3999 g

^4^LODM: Newborn born from DM mothers and birth weight less 2500 g

^a^Analysis by χ^2^ test statistics for 4 groups

^b^Analysis by χ^2^ test statistics for non-DM and HODM groups; NS: Not Significant; *P < 0.001

**Fig. 1:**
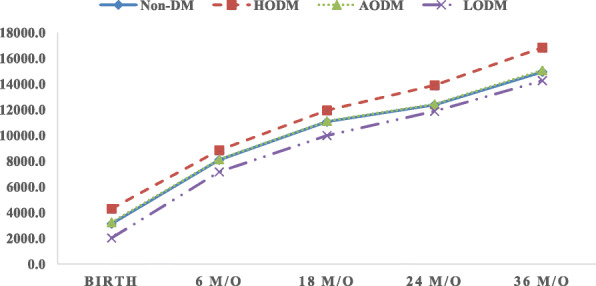
Growth in body weight of children in 4 groups. ^a^Repeated measure ANOVA test for trend among 4 groups; *P < 0.0001. ^b^Cochran–Armitage test for trend among non-DM and HODM groups; *P < 0.05

Analysis of variance (ANOVA) in combination with the Tukey honestly significant difference post hoc test was conducted on normally distributed variables to compare children’s body weight at different time points (birth, 6, 18, 24, and 36 m/o) and perform a sensitive assessment of deviations in growth (Table 2). Figure 1 plots the body weights and changes in growth of all children born in the non-DM and HODM groups from birth to 36 m/o as a result of a Cochran–Armitage test for trend. Separate models for the HODM and non-DM groups were developed to detect influential factors of perinatal conditions, maternal socio-demographic characteristics, infant care, and maternal body weight characteristics. Multiple logistic regression analyses were used to estimate odds ratios (ORs) and 95% confidence intervals for the associations of influential variables between the HODM and non-DM groups at different ages, including maternal BMI, gestational weight gain, breastfeeding, and perinatal conditions (Table 3). The non-DM group was used as the reference group, and *P* < 0.05 indicated statistical significance. Statistical analyses were performed using SAS 9.4. (SAS Institute Inc., Carrie, North Carolina, USA).

**Table 2:** Infant growth from birth to 36 m/o in the HODM and non-DM groups

	HODM	Non-DM	Z value^a^	*P* value^b^
**Birth weight (g)**			0.0028	0.0020
Birth	4294.5 ± 272.8	3122.7 ± 424.3		
6 months	8848.6 ± 1120.8	8090.5 ± 1021.4		
18 months	11,948.4 ± 1346.8	11,062.9 ± 1332.0		
24 months	13,900.0 ± 2004.1	12,373.3 ± 1723.2		
36 months	16,830.8 ± 2500.2	14,939.4 ± 2190.7		
**Weight gain (g)**			0.0029	0.0020
0–6 months	4555.6 ± 1193.0	4965.8 ± 944.6		
0–18 months	7683.4 ± 1380.2	7933.7 ± 1251.8		
0–24 months	9657.9 ± 1984.1	9247.2 ± 1654.3		
0–36 months	12,536.3 ± 2511.6	11,816.0 ± 2118.1		

^a^Z value: Z test statistics

^b^Repeated measure ANOVA

**Table 3:** Multiple logistic regression models of newborn care and maternal body weight factors with childhood overweight

	6 m/o		18 m/o		24 m/o		36 m/o	
	OR	*P*-value	OR	*P*-value	OR	*P*-value	OR	*P*-value
**Null model** **HODM vs. non-DM**	3.95	<0.001	3.32	<0.001	3.25	0.0013	3.74	<0.001
**Model A** **HODM vs. non-DM**	3.70	<0.001	2.98	0.002	3.03	0.004	3.40	<0.001
**Model B** **HODM vs. non-DM**	3.69	<0.001	2.92	0.003	2.96	0.005	3.34	<0.001
Breast feeding at 6 m/o	0.99	NS	0.96	<0.001	0.95	<0.001	0.96	<0.0001
Main caregiver at daytime (mother vs. others)	1.07	NS	1.07	NS	1.35	<0.001	1.02	NS
Non-staple foods feeding before 6 m/o	1.06	NS	1.17	NS	1.12	NS	1.14	0.033
**Model C** **HODM vs. non-DM**	3.19	<0.001	2.27	0.026	2.26	0.045	2.61	0.005
Gestational weight gain (<10 kg vs. 10–14 kg)	0.84	0.003	0.93	NS	0.89	NS	0.83	0.002
Gestational weight gain (>14 kg vs. 10–14 kg)	1.12	0.008	1.22	<0.001	1.25	<0.001	1.24	<0.001
Pre-pregnancy BMI (<18.5 vs. 18.5–25 kg/m^2^)	0.85	0.003	0.77	<0.001	0.63	<0.001	0.68	<0.001
Pre-pregnancy BMI (>25 vs. 18.5–25 kg/m^2^)	1.26	<0.001	1.34	<0.001	1.24	0.011	1.44	<0.001
Postpartum BMI at 6 m/o (<18.5 vs. 18.5–25 kg/m^2^)	1.13	NS	0.88	NS	0.79	NS	1.37	NS
Postpartum BMI at 6 m/o (>25 vs. 18.5–25 kg/m^2^)	1.17	0.002	1.28	<0.001	1.25	0.002	1.31	<0.001
**Model D** **HODM vs. non-DM**	3.17	<0.001	2.22	0.031	2.19	0.055^a^	2.56	0.006
Breast feeding at 6 m/o	0.99	NS	0.96	<0.001	0.95	<0.001	0.96	<0.001
Main caregiver at daytime (mother vs. others)	1.06	NS	1.06	NS	1.35	<0.001	1.03	NS
Non-staple foods feeding before 6 m/o	1.06	NS	1.16	NS	1.09	NS	1.11	NS
Gestational weight gain (<10 kg vs. 10–14 kg)	0.84	0.002	0.93	NS	0.89	NS	0.83	0.0014
Gestational weight gain (>14 kg vs. 10–14 kg)	1.12	0.010	1.22	<0.001	1.25	<0.001	1.23	<0.001
Pre-pregnancy BMI (<18.5 vs. 18.5–25 kg/m^2^)	0.85	0.003	0.76	<0.001	0.62	<0.001	0.67	<0.001
Pre-pregnancy BMI (>25 vs. 18.5–25 kg/m^2^)	1.25	<0.001	1.34	<0.001	1.23	NS	1.45	<0.001
Postpartum BMI at 6 m/o (<18.5 vs. 18.5–25 kg/m^2^)	0.99	NS	0.96	<0.001	0.95	<0.001	0.96	<0.001
Postpartum BMI at 6 m/o (>25 vs. 18.5–25 kg/m^2^)	1.17	0.002	1.27	<0.001	1.26	0.002	1.31	<0.001

Model A: adjustment for perinatal conditions (mode of delivery, gestational age, APGAR score at 1 and 5 min), and maternal socio-demographic characteristics; Model B: adjustment for factors included in model A and newborn care factors (only the ORs of newborn factors are shown); Model C: adjustment for factors included in model A and maternal body weight factors (only the ORs of maternal body weight factors are shown); Model D: adjustment for all factors (only the ORs of newborn care and maternal body weight factors are shown)

*NS* no significance

^a^Borderline significance

## Results

A total of 20,645 (85.3%) pairs of mothers and children were enrolled into our study. Among the pairs, 446 (2.16%) children were born to mothers with diagnosed DM during pregnancy. Regardless of birth weight, all selected influential categories, including perinatal conditions, maternal socio-demographic characteristics, and maternal body weight characteristics, revealed significant differences between the non-DM group and ODM groups, except infant care factors. Compared with mothers without DM, mothers with DM were more likely to have advanced maternal age and live in urban areas. DM was less likely in immigrant women and those with higher education levels (*P* < 0.05). Differences in family income, main caregiver, and breastfeeding versus staple-food feeding at 6 m/o were non-significant. More adverse birth outcomes, such as preterm labor, lower APGAR scores 1 and 5 min after birth, and increased prevalence of birth defects, were present in infants in the ODM groups. Maternal BMIs before and after pregnancy were higher in the DM groups than in the non-DM group. In particular, mothers in the HODM group had obviously higher prevalence of high prepregnancy BMI (46.2% vs. 9.7%) and postpartum BMI (100.0% vs. 63.8%), (*P* < 0.001). Differences in maternal gestational weight gain among the four groups were nonsignificant (*P* = 0.157) (Table 1).

**Fig. 2:**
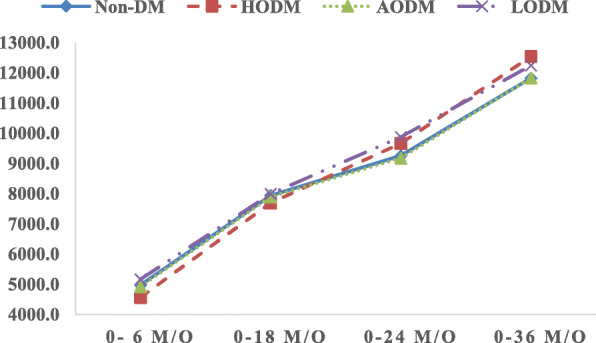
Body weight gain of children in 4 groups. ^a^Repeated measure ANOVA test for trend among 4 groups; *P < 0.0001. ^b^Cochran–Armitage test for trend among non-DM and HODM groups; *P < 0.001

**Fig. 3:**
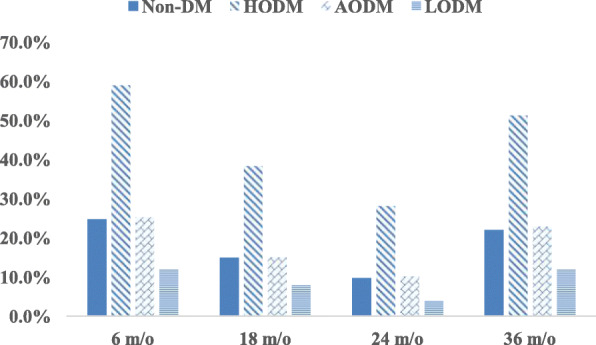
Percentage of OWOB among children in 4 groups at different ages. ^a^χ^2^ test; *P < 0.001

Figure 2 plots the growth of all children in the HODM and non-DM groups. The average birth weight in these two groups was 4294.5 and 3122.7 g, respectively. From birth to 18 m/o, child growth in both the groups HODM had similar velocity and slope in terms of body weight, and average body weight was higher in the non-DM group. However, the velocity of growth in body weight was faster in the HODM group between 18 and 36 m/o. Average body weight at 36 m/o was 16,830.8 and 14,939.4 g in the HODM and non-DM groups, respectively. Growth trends in both the groups were significantly different (*P* < 0.05). Figure 3 presents the percentages of childhood OWOB in the four groups. Children in the HODM group had higher rates of child OWOB at each age stage, especially at 36 m/o.

The disparities in infant body weight and body weight gain between the HODM and non-DM groups at birth, 6, 18, 24, and 36 m/o are shown in Table 2. Children in the HODM group had a higher average body weight than did the non-DM group at different age stages. Body weight gain among infants differed significantly between the two groups. Body weight gain was slower in the HODM group than in the non-DM group before 18 m/o but faster than the non-DM group, from 18 to 36 m/o showed latter catch-up in HODM group, particularly after 24 m/o.

Table 3 presents the results of the stepwise model analysis by using a multiple logistic regression. In the null model, the incidence of OWOB in children in the HODM group was greater than that in children in the non-DM group from birth to 36 m/o (OR: 3.95–3.74). Model A, which adjusted for maternal socio-demographic and perinatal conditions, yielded similar results. When infant care factors in model B were considered, breastfeeding to 6 m/o reduced childhood OWOB among children in the HODM group by approximately 4%, however, mothers as the main daytime caregiver or early staple-food feeding did not affect childhood OWOB (*P* > 0.05). Model C adjusted for the factors included in model A and maternal body weight factors to analyze the effects of maternal body weight on childhood OWOB. Compared with the non-DM group, mothers in the HODM group were more likely to have OWOB children when their gestational weight gain was >14 kg (OR: 1.12–1.25). Obesity (BMI > 25 kg/m^2^) before and after pregnancy also increased the risk of childhood OWOB (OR: 1.24–1.44, 1.17–1.31, respectively). When we adjusted for all potential determinants in model D, maternal DM remained a major risk factor for childhood OWOB at different ages in the HODM group (OR: 2.19–3.17). Maternal body weight before and after pregnancy was a main factor affecting childhood OWOB, and the risk of childhood OWOB increased with the children’s age.

## Discussion

GDM prevalence in Asia has been reported to range from 0.7 to 51.0% [15]. A meta-analysis yielded a pooled GDM prevalence rate of 11.5% [16]. This vast disparity in prevalence rates may be due to differences in diagnostic criteria [17], screening strategies [18], and ethnicity and population characteristics [19]. Our study included 446 mothers with DM who accounted for approximately 2.2% of all included participants. The TBCS study defines whether a mother has DM during pregnancy, in addition to the self-recognition from the interviewees, must be judged by trained interviewers (mainly public health nurses) to determine whether there is “diagnosed DM by doctors during pregnancy”, and refer to the check-up records on the maternal health booklet issued by Taiwan’s National Health Insurance (NHI). The coverage rate of NHI is >99%. Pregnant women can receive at least 10 prenatal health examinations by obstetricians, and barriers in access to prenatal care are lower in Taiwan compared with those in other countries. This discrepancy in prevalence rates might be related to the design of the TBCS, which was connected as a self-report questionnaire by mothers administered in interviews. Although the urine glucose level is a routine parameter tested during prenatal examinations, serum glucose levels and oral glucose tolerance tests are not routine items designated by Taiwan’s NHI for prenatal examinations, mothers with DM may not be aware of their DM status. Such issue may cause some GDM mothers not being diagnosed and not included in DM group of this study. However, it is reported that nearly 95% of pregnant women received free prenatal care by the NHI in Taiwan. Doctors are required to screen for maternal DM by NHI guidelines, so we believe it has consistent validity. Our study using a dataset from the TBCS is the first to use a multistage stratified systematic sampling design, and the content of the questionnaire covered a wide range of information. TBSC has been widely applied in research to reveal insights into the health profiles of children growing up in Taiwan.

Advanced maternal age, family history of DM, ethnicity, overweight or obesity, and smoking are well-documented risk factors for GDM [20]. In addition to these risk factors, data increasingly indicate that diet and lifestyle factors both before and during pregnancy are associated with GDM [21]. A study in Taiwan indicated that advanced maternal age, a family history of DM, a less than high school education, high prepregnancy BMI, and an indigenous ethnicity were risk factors for GDM [22]. In our study, mothers with DM were older, lived in urban areas, were less likely to be immigrants, and had higher education levels (*p* < 0.05). Their BMIs before pregnancy and postpartum at 6 months were higher than those of the non-DM group. In particular, mothers in the HODM group had higher prepregnancy BMIs. The disparity in maternal age may explain why mothers with DM had higher education levels; moreover, immigrant and indigenous mothers in Taiwan are relatively younger.

Because of the potential confounders including genetic factors, mother and infant lifestyle, and maternal obesity factors are difficult to control, the relationships between maternal DM and risk of childhood OWOB are complex. The Taiwan’s NHI has a high coverage rate and people face few barriers to medical assessments. We focused on the HODM group because mothers in the LODM group tended to have pregestational DM that increased the incidence of congenital anomalies and preterm labor. Our results indicate that maternal obesity before and after pregnancy increase the risk of childhood OWOB, and this risk increases as children age. These findings suggest that genetics may play a central role. A previous study identified many genetic polymorphisms in genome-wide association studies of adult BMI and the genetic susceptibility to childhood obesity; the association was partially explained by appetitive traits in infancy followed by an early childhood increase in BMI [23].

Studies of the effects of gestational DM on childhood overweight and obesity have yielded inconclusive results. A meta-analysis reported inconsistent evidence of an association of GDM with childhood overweight and obesity because of methodological limitations in the included studies [9]. However, our study adjusted for prepregnancy obesity, infant care, and maternal socio-demographic factors. Maternal DM was an independent determinant of childhood OWOB, but the associations were attenuated after adjustment for prepregnancy and postpartum maternal BMI. Previous studies have not focused on infants with macrosomia, which may have confounded their results. Because the infants of mothers with DM, particularly type 1 DM, have a higher risk of congenital anomalies, premature birth, and low APGAR scores, their growth is expected to be slower than that of infants of mothers without DM or even mothers with GDM. We conducted a stepwise logistic regression by using five models to eliminate these confounders.

Because of mothers with overweight or obesity have a higher prevalence of DM, our study could not identify the prevalence of DM in ODM children. Mothers with obesity may have a genetic predisposition to have children who become overweight; in addition, maternal lifestyle, diet, and maternal cognition to infant body weight are factors of childhood overweight [24]. In our study, main daytime caregiver and early staple-food feeding were not influential factors in Taiwanese cohort. However, high calorie intake in infancy and large appetite from 1 year were reported to be related to a higher incidence of subsequent childhood obesity [23]. Children who were breastfed until 12 m/o were reported to be 2.7 times less likely to develop childhood obesity [24, 25]. We demonstrated that breastfeeding until 6 m/o is a protective factor against childhood OWOB (OR: 0.96). Although the benefits of breastfeeding appear to be a key part of the positive health outcomes associated with the parent-child relationship, the effect of the dose is not informed in this study. The odds ratio of HODM group to the non-DM group for childhood OWOB is range from 3.25 to 3.95 at different age stages. Regardless of whether neonatal care factors were adjusted in Model A or Model B, the odds of HODM in the probability of children being OWOB has similar findings. The effects to the odds of HODM between the former two are much lower than those of Model C and Model D. Therefore, we infer that HODM is an independent factor affecting childhood OWOB, and the impact of maternal overweight or obesity is much greater than that of perinatal conditions and acquired care. Control of DM and restricted weight gain during pregnancy are keys to preventing childhood obesity, especially in mothers with DM.

The strength of our study is its focus on a particular infant population for comparison with children whose mothers did not have DM and its exclusion of infants with low birth weight and congenital anomalies to eliminate the effect of congenital or genetic factors that could confound the statistical results. We also used a stepwise logistic regression model to detect confounders and their interactions between DM and diabetes-related perinatal complications. In addition, we analyzed prepregnancy and postpartum BMI to compare the effects of maternal BMI and gestational body weight gain with childhood OWOB. Moreover, this cohort study can offer information on the determinants of childhood OWOB at different ages.

Our research has some limitations. The design of the TBCS questionnaires did not differentiate between pregestational DM and GDM, neither lack of medical records about the severity of hyperglycemia and related medical intervention. This limitation makes genetic factors and the effects of DM control difficult to identify. Another limitation is the high rate of cesarean deliveries in Taiwan; some infants with macrosomia can be delivered earlier if their body weight is estimated to be overweight during prenatal examinations. This phenomenon may have reduced the number of infants in the HODM group. The maternal height and weight were self-reported instead of measurements might latent errors. This cohort study followed children to 36 m/o. Future studies may prolong the follow-up period to analyze the long-term relationships between mothers with DM and childhood obesity.

## Conclusion and suggestions

In this population-based cohort study, maternal DM and BMI of mothers more than 25 kg/m^2^ prepregnancy and postpartum were the significant factors of childhood OWOB, and the maternal BMI effects increased as children aged. Prolonged breastfeeding to the age of 6 months and weight gain control during pregnancy are protection factors. DM control during pregnancy, restricted maternal gestational body weight gain, maternal BMI control, and prolonged breastfeeding are strategies for mothers with DM to prevent childhood overweight.

## Data Availability

Please contact to Meng-Chih Lee, E-Mail: mengchihlee@gmail.com

## Abbreviations

AODM: Appropriate birth weight Of mothers with Diabetic Mellitus
BMI: Body Mass Index
GDM: Gestational Diabetic Mellitus
HODM: High birth weight children Of mothers with Diabetic Mellitus
LODM: Low birth weight Of mothers with Diabetic Mellitus
MCS: Millennium Cohort Study
NCS: National Children’s Study
NHI: National Health Insurance
ODM: Offspring of mother with Diabetic Mellitus
OWOB: Overweight/Obesity
TBCS: Taiwan Birth Cohort Study

## References

[1] Statistics Department of the Ministry of the Interior. Annual report on internal affairs statistics: fertility rate of women of childbearing age 2018. https://moi.gov.tw/files/site_stuff/321/2/year/year.html.

[2] Huang YC, Fang LR, Huang JP, Xu SB, Zhu CH (2005). Investigation of gestational diabetes in Taiwan. Taipei Med J.

[3] Chou CY, Lin CL, Yang CK, Yang WC, Lee FK, Tsai MS (2010). Pregnancy outcomes of Taiwanese women with gestational diabetes mellitus: a comparison of Carpenter-Coustan and National Diabetes Data Group criteria. J Women’s Health.

[4] Aldana-Parra F, Vega GO, Fewtrell M (2020). Associations between maternal BMI, breastfeeding practices and infant anthropometric status in Colombia; secondary analysis of ENSIN 2010. BMC Public Health.

[5] Chu SY, Callaghan WM, Kim SY, Schmid CH, Lau J, England LJ (2007). Maternal obesity and risk of gestational diabetes mellitus. Diabetes Care.

[6] Burke V, Beilin LJ, Dunbar D (2001). Family lifestyle and parental body mass index as predictors of body mass index in Australian children: a longitudinal study. Int J Obes.

[7] Cartagena D, McGrath JM, Masho SW (2016). Differences in modifiable feeding factors by overweight status in Latino infants. Appl Nurs Res.

[8] Brown CL, Skinner AC, Yin HS, Rothman RL, Sanders LM, Delamater AM (2016). Parental perceptions of weight during the first year of life. Acad Pediatr.

[9] Kim SY, England JL, Sharma JA, Njoroge T (2011). Gestational diabetes mellitus and risk of childhood overweight and obesity in offspring: a systematic review. Exp Diabetes Res.

[10] Svensson V, Jacobsson JA, Fredriksson R, Danielsson P, Sobko T, Schiöth HB (2011). Associations between severity of obesity in childhood and adolescence, obesity onset and parental BMI: a longitudinal cohort study. Int J Obes.

[11] Chiang TL, Lin SJ, Chang MC. Taiwan Birth Cohort Study: backgrounds, design and participants. Taiwan Health Promotion Administration, MOHW; 2009 https://www.hpa.gov.tw/Pages/List.aspx?nodeid=110.

[12] Chesbrough KB, Ryan MA, Amoroso P, Boyko EJ, Gackstetter GD, Hooper TI (2002). The Millennium Cohort Study: a 21-year prospective cohort study of 140,000 military personnel. Mil Med.

[13] Landrigan PJ, Trasande L, Thorpe LE, Gwynn C, Lioy PJ, D’Alton ME (2006). The national children’s study: a 21-year prospective study of 100 000 American children. AAP.

[14] Chen W, Chang M-H (2010). New growth charts for Taiwanese children and adolescents based on World Health Organization standards and health-related physical fitness. Pediatr Neonatol.

[15] Nguyen CL, Pham NM, Binns CW, Duong DV, Lee AH (2018). Prevalence of gestational diabetes mellitus in eastern and southeastern Asia: a systematic review and meta-analysis. J Diabetes Res.

[16] Lee KW, Ching SM, Ramachandran V, Yee A, Hoo FK, Chia YC (2018). Prevalence and risk factors of gestational diabetes mellitus in Asia: a systematic review and meta-analysis. BMC Pregnancy Childbirth.

[17] Lauring JR, Kunselman AR, Pauli JM, Repke JT, Ural SH (2018). Comparison of healthcare utilization and outcomes by gestational diabetes diagnostic criteria. J Perinat Med.

[18] Corrado F, Pintaudi B (2017). Diagnosis of gestational diabetes mellitus: Italian perspectives on risk factor-based Screening. Nutr Diet Mater Diabet.

[19] Alfadhli EM, Osman EN, Basri TH, Mansuri NS, Youssef MH, Assaaedi SA (2015). Gestational diabetes among Saudi women: prevalence, risk factors and pregnancy outcomes. Ann Saudi Med.

[20] Ben-Haroush A, Yogev Y, Hod M (2004). Epidemiology of gestational diabetes mellitus and its association with Type 2 diabetes. Diabet Med.

[21] Zhang C, Ning Y (2011). Effect of dietary and lifestyle factors on the risk of gestational diabetes: review of epidemiologic evidence. Am J Clin Nutr.

[22] Lin PC, Hung CH, Chan TF, Lin KC, Hsu YY, Tzeng YL (2016). The risk factors for gestational diabetes mellitus: a retrospective study. Midwifery.

[23] de Lauzon-Guillain B, Koudou YA, Botton J, Forhan A, Carles S, Pelloux V (2019). Association between genetic obesity susceptibility and mother-reported eating behaviour in children up to 5 years. Pediatr Obes.

[24] Reifsnider E, McCormick DP, Cullen KW, Todd M, Moramarco MW, Gallagher MR (2018). Randomized controlled trial to prevent infant overweight in a high-risk population. Acad Pediatr.

[25] Sun J, Wu L, Zhang Y, Li C, Wang Y, Mei W (2020). Association of breastfeeding duration, birth weight, and current weight status with the risk of elevated blood pressure in preschoolers. Eur J Clin Nutr.

